# Space-frequency-polarization-division multiplexed wireless communication system using anisotropic space-time-coding digital metasurface

**DOI:** 10.1093/nsr/nwac225

**Published:** 2022-10-18

**Authors:** Jun Chen Ke, Xiangyu Chen, Wankai Tang, Ming Zheng Chen, Lei Zhang, Li Wang, Jun Yan Dai, Jin Yang, Jun Wei Zhang, Lijie Wu, Qiang Cheng, Shi Jin, Tie Jun Cui

**Affiliations:** State Key Laboratory of Millimeter Waves, Southeast University, Nanjing210096, China; Institute of Electromagnetic Space, Southeast University, Nanjing210096,China; Frontiers Science Center for Mobile Information Communication and Security, Southeast University, Nanjing210096, China; National Mobile Communications Research Laboratory, Southeast University, Nanjing210096, China; National Mobile Communications Research Laboratory, Southeast University, Nanjing210096, China; State Key Laboratory of Millimeter Waves, Southeast University, Nanjing210096, China; Institute of Electromagnetic Space, Southeast University, Nanjing210096,China; Frontiers Science Center for Mobile Information Communication and Security, Southeast University, Nanjing210096, China; State Key Laboratory of Millimeter Waves, Southeast University, Nanjing210096, China; Institute of Electromagnetic Space, Southeast University, Nanjing210096,China; Frontiers Science Center for Mobile Information Communication and Security, Southeast University, Nanjing210096, China; State Key Laboratory of Millimeter Waves, Southeast University, Nanjing210096, China; Institute of Electromagnetic Space, Southeast University, Nanjing210096,China; Frontiers Science Center for Mobile Information Communication and Security, Southeast University, Nanjing210096, China; State Key Laboratory of Millimeter Waves, Southeast University, Nanjing210096, China; Institute of Electromagnetic Space, Southeast University, Nanjing210096,China; Frontiers Science Center for Mobile Information Communication and Security, Southeast University, Nanjing210096, China; Southeast University Wuxi Campus, Wuxi214061, China; State Key Laboratory of Millimeter Waves, Southeast University, Nanjing210096, China; Institute of Electromagnetic Space, Southeast University, Nanjing210096,China; Frontiers Science Center for Mobile Information Communication and Security, Southeast University, Nanjing210096, China; State Key Laboratory of Millimeter Waves, Southeast University, Nanjing210096, China; Institute of Electromagnetic Space, Southeast University, Nanjing210096,China; Frontiers Science Center for Mobile Information Communication and Security, Southeast University, Nanjing210096, China; State Key Laboratory of Millimeter Waves, Southeast University, Nanjing210096, China; Institute of Electromagnetic Space, Southeast University, Nanjing210096,China; Frontiers Science Center for Mobile Information Communication and Security, Southeast University, Nanjing210096, China; National Mobile Communications Research Laboratory, Southeast University, Nanjing210096, China; State Key Laboratory of Millimeter Waves, Southeast University, Nanjing210096, China; Institute of Electromagnetic Space, Southeast University, Nanjing210096,China; Frontiers Science Center for Mobile Information Communication and Security, Southeast University, Nanjing210096, China

**Keywords:** polarization-division multiplexing, frequency-division multiplexing, space-division multiplexing, space-time coding, wireless communication

## Abstract

In the past few years, wireless communications based on digital coding metasurfaces have gained research interest owing to their simplified architectures and low cost. However, in most of the metasurface-based wireless systems, a single-polarization scenario is used, limiting the channel capacities. To solve the problem, multiplexing methods have been adopted, but the system complexity is inevitably increased. Here, a space-frequency-polarization-division multiplexed wireless communication system is proposed using an anisotropic space-time-coding digital metasurface. By separately designing time-varying control voltage sequences for differently oriented varactor diodes integrated on the metasurface, we achieve frequency-polarization-division multiplexed modulations. By further introducing different time-delay gradients to the control voltage sequences in two polarization directions, we successfully obtain space-frequency-polarization-division multiplexed modulations to realize a wireless communication system with a new architecture. The new communication system is designed with compact dual-polarized meta-elements, and can improve channel capacity and space utilization. Experimental results demonstrate the high-performance and real-time transmission capability of the proposed communication system, confirming its potential application in multiple-user collaborative wireless communications.

## Teaser

A new-architecture space-frequency-polarization-division multiplexed wireless communication system is proposed using an anisotropic space-time-coding digital metasurface, which can improve the channel capacity and space utilization.

## INTRODUCTION

The rapid development of modern wireless communication technologies has led to a shortage of spatial and spectral resources, bringing enormous challenges to the field of wireless communications. This issue has promoted the adoption of multiplexing, which is commonly used in computer networks and telecommunications. In the microwave, terahertz and optical regions, signal multiplexing methods include space, angle, polarization, frequency and orbital angular momentum multiplexing [1–6]. In traditional wireless communications, these multiplexing methods require multiple coding algorithms and physical circuits, which make the hardware design and software complicated, and reduce the resource utilization. In addition, as the system becomes more complicated, the overheads for maintaining system synchronization and stability will increase. Therefore, novel wireless communication architectures are required to fully leverage the multiplexing.

Digital coding and programmable metasurfaces provide broad interfaces between digital-information and physical worlds, showing promise for the next generation of smart wireless communications [7–13]. By customizing the electromagnetic (EM) properties of periodically or non-periodically arranged subwavelength meta-atoms using a field-programmable gate array (FPGA), wave propagation and wave-matter interaction can be dynamically manipulated for applications such as hologram imaging, anomalous reflection/refraction and polarization control [7,14–19]. More importantly, the digital coding characterization allows us to map the EM wave parameters to digital information using the coding sequences to support metasurface-based information transmissions [11,20–23]. Further, time-domain digital coding metasurfaces have been proposed to enable non-linear harmonic spectral reconfiguration [24–37], bringing new degrees of freedom for metasurface-based wireless communications. Currently, various metasurface-based wireless communication systems have been realized to directly modulate digital information onto the carrier waves by metasurfaces themselves and transmit the modulated signals to free space [11,21,25,38–46]. This information transmission strategy does not need a radio-frequency (RF) mixer and transmitting antenna, which greatly simplifies the frame of the traditional transmitters and reduces the hardware costs.

However, most of the existing metasurface-based communication systems have only one channel for information transmissions, and the amount of transmitted information is limited. To solve the above problem, a polarization modulation scheme was proposed using a polarization-controllable digital coding metasurface to realize dual-channel communications by shifting the incident frequencies of the metasurface at different time slots [22]. However, such a dual-channel communication scheme requires incident frequencies to change with time, resulting in very low spectrum utilization of the system and great difficulty in integrating the RF front-ends. Besides that, some prototypes of 2 × 2 multiple-input multiple-output (MIMO) wireless transmission systems, based on metasurfaces, have been developed to transmit the information through two channels simultaneously [47,48]. But these efforts only achieved single-frequency signal transmissions, which neglected the potential of space-time-coding (STC) digital metasurfaces to manipulate the signals and beams at the same time. In addition, MIMO transmission requires joint demodulations of multiple received signals, and therefore increases the system complexity. An STC-metasurface wireless communication system based on space- and frequency-division multiplexing has been proposed to transmit multiple information streams to terminals at various locations simultaneously and independently via different frequencies [49]; the key is to optimize the STC matrices in real time to control the harmonic power intensities in different directions. Nevertheless, the design and optimization of the STC matrices will increase the design difficulty of the information modulation, and it will be hard to satisfy the requirements of real-time applications. Besides that, the proposed communication scheme relies on the presence and absence of harmonic energies, which results in low energy utilization of the whole communication system.

To improve the channel capacity and space utilization of the wireless communication systems and reduce the complexity, here we propose a space-frequency-polarization-division multiplexed wireless communication system using an anisotropic STC digital metasurface. Previously, the anisotropic STC digital metasurface has been employed for controlling dual-polarized beams and generating arbitrary polarizations in real time [16,33,34]. In this work, we explore its advanced features in new wireless communication schemes. We firstly present a polarization-division multiplexed (PDM) architecture that can achieve independent signal modulations in two orthogonal directions using the anisotropic STC digital metasurface. To detect and separate different signals and increase the system capacity, we modulate the signals from two polarization channels onto different harmonic waves, achieving frequency-polarization-division multiplexed modulations. Subsequently, time-delay gradients are introduced in the control signals on the rows and columns of the metasurface for dynamic dual-harmonic beamforming along two polarization directions, realizing space-frequency-polarization-division multiplexed modulations. For experimental validations, we create two experiments. We firstly demonstrate independent and synchronous video transmissions based on a frequency-polarization-division multiplexed scheme. Then we further validate the performance of the space-frequency-polarization-division multiplexed scheme along the two polarization directions. The experimental results confirm the applicability of the proposed wireless communication system to multi-user scenarios.

## POLARIZATION-DIVISION MULTIPLEXED ARCHITECTURE USING THE ANISOTROPIC STC DIGITAL METASURFACE

**Figure 1.**
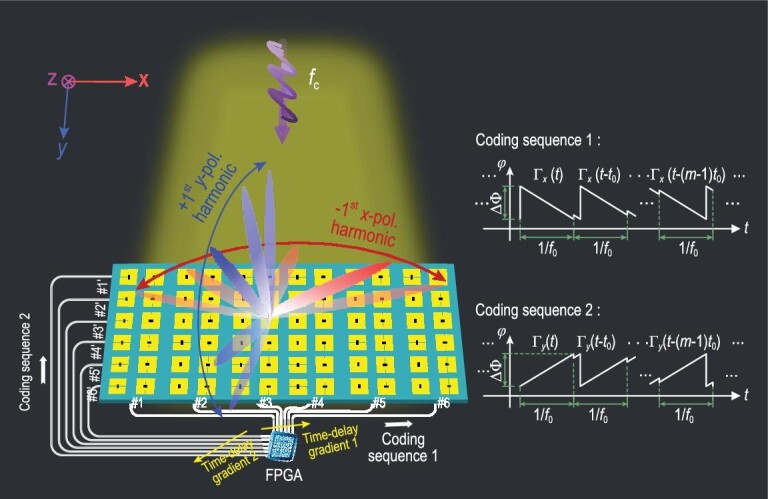
The space-frequency-polarization-division multiplexing architecture using an anisotropic STC digital metasurface for simultaneously scanning harmonic beams at different frequencies on two orthogonal polarizations, which results in a low-cost space-frequency-polarization-division multiplexed wireless communication system. Coding sequences 1 and 2 are the basic time-varying control waveforms, which are used to drive the varactor diodes parallel to the *x* and *y* polarization directions. Different time-delay gradients 1 and 2 are added in the two coding sequences in different control lines with the same polarization.

We present a PDM architecture using the anisotropic STC digital metasurface, as illustrated in Fig. 1. Each metasurface element is composed of two parts that independently control the *x*- and *y*-polarized EM properties by sharing the aperture. A linearly polarized plane wave radiated by a feed source, whose polarization direction is not parallel to both *x* and *y* axes (e.g. a 45° linearly polarized wave), serves as the incident wave to normally illuminate the metasurface. Owing to the anisotropic EM property, the metasurface can decompose the incident electric-field vector into two orthogonal components, thereby obtaining *x-* and *y*-polarized branches. Due to their inherent orthogonality, two independent reflected waves are generated simultaneously by the anisotropic STC digital metasurface. If the reflection coefficients (including their amplitudes and phases) in the two polarization directions can be adjusted in real time, we can realize independent controls of the amplitude and phase spectra for the *x-* and *y-*polarized reflected waves, along with independent signal modulations for a pair of orthogonally polarized EM waves. Hence, we can perform the PDM signal transmission using the anisotropic STC digital metasurface.

The dual-polarized reflection property of the anisotropic STC digital metasurface can be described by Jones matrix ${\boldsymbol{H}}( {\boldsymbol{t}} )$, which is a 2 × 2 complex matrix expressing the relationship between the two orthogonal electric-field components in the time domain at the incidence and reflection [33]:

(1) \begin{equation*} {\boldsymbol{H}}({\boldsymbol{t}}) = \left[ {\begin{array}{@{}*{2}{c}@{}} {{\Gamma}_{\!\! xx}(t)}&\quad{{\Gamma}_{\!\! xy}(t)}\\ {{\Gamma}_{\!\! yx}(t)}&\quad {{\Gamma}_{\!\! yy}(t)} \end{array}} \right], \end{equation*}

where ${\Gamma}_{\!\! xx}$ and ${\Gamma}_{\!\! yy}$ are co-polarized reflection coefficients for the *x* and *y* polarizations, respectively. Assuming that the metasurface elements have extremely high cross-polarization isolation, the cross-polarized reflection coefficients can be simplified as ${\Gamma}_{\!\! xy} = {\Gamma}_{\!\! yx} = 0$. We express ${\Gamma}_{\!\! xx}$ and ${\Gamma}_{\!\! yy}$ in phasor notations, then ${\boldsymbol{H}}{({\boldsymbol{t}})}$ is rewritten as

(2) \begin{equation*} {\boldsymbol{H}}({\boldsymbol{t}}) = \left[ {\begin{array}{@{}*{2}{c}@{}} {| {{\Gamma}_{\!\! xx}(t)} |{e}^{j{{\rm{\Phi }}}_{\!\! xx} (t)}}&\quad 0\\ 0&\quad {| {{\Gamma}_{\!\! yy} (t)}|{e}^{j{{\rm{\Phi}}}_{\!\! yy} (t)}} \end{array}} \right],\end{equation*}

where $| {\Gamma ( t )} |$ and ${\rm{\Phi }}( t )$ are the amplitude and phase of $\Gamma ( t )$, respectively. Hence, the dual-polarized reflection process can be characterized by

(3) \begin{equation*} {{\boldsymbol{E}}}_{\boldsymbol{\!\! r}}({\boldsymbol{t}}) = {\boldsymbol{H}}({\boldsymbol{t}}) \cdot {{\boldsymbol{E}}}_{\boldsymbol{\!\! i}}({\boldsymbol{t}}). \end{equation*}

Both notation vectors ${{\boldsymbol{E}}}_{\boldsymbol{\!\! i}}( {\boldsymbol{t}} )$ and ${{\boldsymbol{E}}}_{\boldsymbol{\!\! r}}( {\boldsymbol{t}} )$ consist of two elements, indicating the two polarization components:

$$\begin{eqnarray*}
{{\boldsymbol{E}}}_{\boldsymbol{\!\! i}}( {\boldsymbol{t}} ) = \left[ {\begin{array}{@{}*{1}{c}@{}} {{E}_{\!\! ix}( t )}\\ {{E}_{\!\! iy}( t )} \end{array}} \right]
\end{eqnarray*}$$

and

$$\begin{eqnarray*}
{{\boldsymbol{E}}}_{\boldsymbol{\!\! r}}( {\boldsymbol{t}} ) = \left[ {\begin{array}{@{}*{1}{c}@{}} {{E}_{\!\! rx}( t )}\\ {{E}_{\!\! ry}( t )} \end{array}}\right].
\end{eqnarray*}$$

Since Equation (3) is similar to the reflection relation in the single-polarized mode [20,21,25,40–44,47,49], the existing metasurface-based wireless communication systems can be easily adapted to the PDM operation.

## FREQUENCY-POLARIZATION-DIVISION MULTIPLEXED MODULATIONS BASED ON THE ANISOTROPIC STC DIGITAL METASURFACE

From Equation (2), it is clear that the simultaneous and independent control of the time-varying co-polarized reflection coefficients ${\Gamma}_{\!\! xx}$ and ${\Gamma}_{\!\! yy}$ is the basis for realizing PDM wireless communications. According to the principle of metasurface-based communication [20,40], we can simultaneously modulate the signals (both amplitude and phase) on each polarization channel by establishing the relation between the digital symbols and reflection coefficients of the metasurface. Moreover, considering the mapping relation in refs. [25,41,47] and adopting a series of time-varying reflection coefficients as the symbols in different time slots, we can realize PDM frequency modulation, PDM harmonic amplitude/phase modulations and even more sophisticated high-order PDM signal modulations.

**Figure 2.**
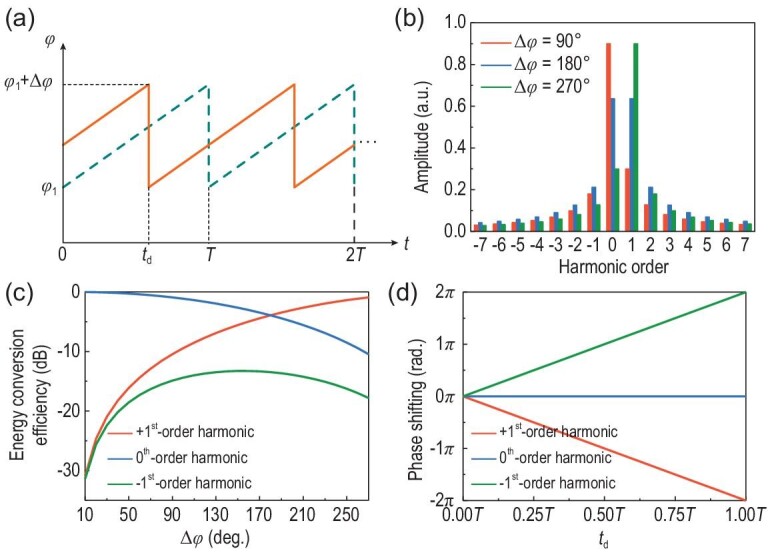
(a) Time-varying phases of the original (dashed line) and time-delayed (solid line) reflection coefficients. (b) Calculated harmonic amplitude distributions with phase differences ($\Delta \varphi $) of 90°, 180° and 270°, respectively. (c) Calculated energy conversion efficiencies of the +1st, 0th and −1st harmonics as the functions of $\Delta \varphi $. (d) Calculated phase shifting of the +1st, 0th and −1st harmonics as the functions of time delay ${t}_d$.

To separate multiple signals from different polarization channels, simplify independent multi-stream demodulations at the receiver end, and improve the system capacity, we propose a frequency-polarization-division multiplexed modulation scheme to carry two differently polarized signal streams through two carrier waves at different frequencies. For example, we consider frequency-polarization-division multiplexed multiple phase-shift keying (MPSK) modulation for two polarized receivers through harmonic frequencies *f*_c_ − *f*_0_ and *f*_c_ + *f*_0_. For efficient harmonic signal modulation at a fixed polarization, a type of time-varying reflection coefficient $\Gamma ( t )$ with linearly varied phase over time is used, as shown in Fig. 2a. Its instantaneous expression in one period is expressed as

(4) \begin{equation*} \Gamma (t) = A{e}^{j({{\varphi}_1 + \frac{{\Delta \varphi }}{T} \cdot t})},\quad 0 \le t \le T, \end{equation*}

where *A* is the magnitude of $\Gamma ( t )$, *T* is the period of the symbol and ${\varphi }_1$ and $\Delta \varphi $ are the initial phase state and phase difference, respectively. For the reflection-type metasurface with low return loss, *A* can be regarded as a constant at any time *t*. Under the normal radiation of the monochromatic plane wave ${E}_{\! i}( t ) = {e}^{j{\omega }_ct}$ (where ${\omega }_c = 2\pi {f}_c$ is the angular frequency of the incident wave), the reflected wave ${E}_{\! r}( \omega )$ will be distributed at harmonic frequencies [29]. The derivation of the specific harmonic amplitude/phase distribution is available in Supplementary Note 1. Figure 2b gives the harmonic amplitude distributions with different phase differences $\Delta \varphi $, and Fig. 2c shows the energy conversion efficiencies of the +1st, 0th and −1st harmonics according to $\Delta \varphi $ with the reflection amplitude *A* = 1. We observe that more efficient signal modulations at the +1st harmonic can be achieved by increasing $\Delta \varphi $. On the contrary, if $\Delta \varphi $ is negative and $\Delta \varphi < - \pi $, as $\Delta \varphi $ decreases, the signal modulation is the most efficient at the −1st harmonic. Therefore, the reflection function ${\boldsymbol{H}}{({\boldsymbol{t}} )}$ of the meta-atom for frequency-polarization-division multiplexed signal modulations in a period can be expressed as

(5) \begin{equation*} {\boldsymbol{H}}({\boldsymbol{t}}) = \left[ {\begin{array}{@{}*{2}{c}@{}} {{A}_x{e}^{j\Delta \varphi ({1 - \frac{t}{T}})}}&\ \ 0\\ 0&\ \ {{A}_y{e}^{j\Delta \varphi \frac{t}{T}}} \end{array}} \right],\ \ 0 \le t \le T, \end{equation*}

where ${A}_x$ and ${A}_y$ represent the co-polarized reflection amplitudes along the *x* and *y* directions, respectively. Hence, the *x*- and *y*-polarized signal modulations can be implemented at the frequencies *f*_c_ − *f*_0_ and *f*_c_ + *f*_0_, respectively.

To perform the MPSK modulations at the desired harmonics, the harmonic multi-bit phases must be precisely controlled. To this end, we introduce time delay ${t}_d$ into the time-varying reflection coefficient, as shown by the solid line in Fig. 2a. The reflection coefficient with time delay ${t}_d$ over one period can be expressed as

(6) \begin{equation*}\Gamma ({t - {t}_d}) = \left\{ {\begin{array}{@{}*{1}{c}@{}} {A{e}^{j\left[ {{\varphi }_1 + \frac{{\Delta \varphi }}{T} \cdot \left( {t + T - {t}_d} \right)} \right]},\quad 0 \le t \le {t}_d}\\ {A{e}^{j\left[ {{\varphi }_1 + \frac{{\Delta \varphi }}{T} \cdot \left( {t - {t}_d} \right)} \right]},\quad {t}_d < t \le T} \end{array}} \!\! \right..\end{equation*}

Using the Fourier transform $\mathcal{F}[ {\Gamma ( {t - {t}_d} )} ] = {e}^{ - j\omega {t}_d}\Gamma ( {j\omega } )$, we obtain the *k*th harmonic phase increment as $\Delta {\rm{\Phi }}{|}_{{f}_c + k{f}_0} = - k{\omega }_0{t}_d$, while the harmonic energy remains unchanged [29]. Figure 2d shows the phase delays of the +1st, 0th and −1st harmonics according to the time delay ${t}_d$. Therefore, a series of time-delayed reflection coefficients with proper ${t}_d$ in two polarizations enable the frequency-polarization-division multiplexed MPSK modulation. The allocation strategy of the modulation signal sets for the two polarization channels is detailed in Supplementary Note 2. Then, the *x*-polarized MPSK modulation occurs at the −1st harmonic, and the *y*-polarized MPSK modulation occurs simultaneously at the +1st harmonic, thus providing a general paradigm for the rapid design of frequency-polarization-division multiplexed MPSK modulations.

## SPACE-FREQUENCY-POLARIZATION-DIVISION MULTIPLEXED MODULATION WITH WIDE-ANGLE COVERAGE ALONG TWO POLARIZATION DIRECTIONS

Equation (5) describes the reflection function of the meta-atom for frequency-polarization-division multiplexed modulations. If all elements of the metasurface share the same reflection coefficient, the communication can be implemented in a finite region around the normal direction of the metasurface, leading to a low space utilization rate of the communication system. Hence, we expect to implement space-frequency-polarization-division multiplexed modulations along two polarization directions by fully exploiting the harmonic beamforming of the anisotropic STC digital metasurface.

To perform the harmonic beamforming, we need to design the phase profile at the target harmonic [50,51]. As shown in Fig. 2d, an arbitrary harmonic phase profile can be obtained by constructing the corresponding time-delay gradients on the reflection coefficients of all meta-atoms. Consider a general case as an example. We firstly divide the anisotropic STC digital metasurface into *M* rows and *N* columns to independently control the *x*-polarized EM property of the same column, and the *y*-polarized EM property of the same row. Then we separately set independent time-delay gradient functions *tg*1(*x*) and *tg*2(*y*) in the *x* and *y* directions, among the control signals of different columns and rows, as shown in Fig. 1. It must be pointed out that the time-delay gradients *tg*1(*x*) and *tg*2(*y*) are different from the time delay ${t}_d$ of the reflection coefficients for MPSK modulations; the former exists among the different columns and rows, whereas the latter is only present in the different time slots of a specific column or row. Thus, the reflection function ${\boldsymbol{H}}( {\boldsymbol{t}} )$ of each meta-atom can be written as

(7) \begin{eqnarray*} &&{\boldsymbol{H}}({{\boldsymbol{t}},{\boldsymbol{x}},{\boldsymbol{y}}})\nonumber\\ &&\qquad = \left[ {\begin{array}{@{}*{2}{c}@{}} {{\Gamma}_{\!\! x}\left[ {t - tg1(x)} \right]}&\quad 0\\ 0&\quad {{\Gamma }_{\!\! y}\left[ {t - tg2 (y)} \right]} \end{array}} \right],\nonumber\\ &&\qquad\qquad\qquad 0 \le t \le T, \end{eqnarray*}

where ${\Gamma}_{\!\! x}[ {t - tg1( x )} ]$ and ${\Gamma}_{\!\! y}[ {t - tg2( y )} ]$ are given by Equation (6). Referring to the classic antenna array theory, the harmonic phase profiles produced by the time-delay gradients *tg*1(*x*) and *tg*2(*y*) cause the −1st *x*-polarized and +1st *y*-polarized harmonic beams to be deflected to the angle ${\theta}_{\!\! x}$ on the *xoz* plane and ${\theta}_{\!\! y}$ on the *yoz* plane, respectively [35]:

(8a) \begin{equation*}{\theta}_{\! x} = {\textit{arcsin}}\left\{ {\frac{{c{f}_{\! 0}}}{{{f}_{\!c} - {f}_{\!0}}}\frac{{d\left[ {tg1 (x)} \right]}}{{dx}}} \right\},\end{equation*}

(8b) \begin{equation*}{\theta }_{\! y} = {\textit{arcsin}}\left\{ { - \frac{{c{f}_{\! 0}}}{{{f}_{\! c} + {f}_{\! 0}}}\frac{{d\left[ {tg2 (y)} \right]}}{{dy}}} \right\},\end{equation*}

where *c* is the light speed in vacuum, ${f}_0 = \frac{1}{T}$ is the frequency of the control signals and $\frac{{d( \cdot )}}{{dx}}$ and $\frac{{d( \cdot )}}{{dy}}$ denote the derivatives with respect to positions *x* and *y*, respectively. Consequently, dynamic dual-harmonic beamforming along two polarization directions can be performed by independently switching *tg*1(*x*) and *tg*2(*y*).

**Figure 3.**
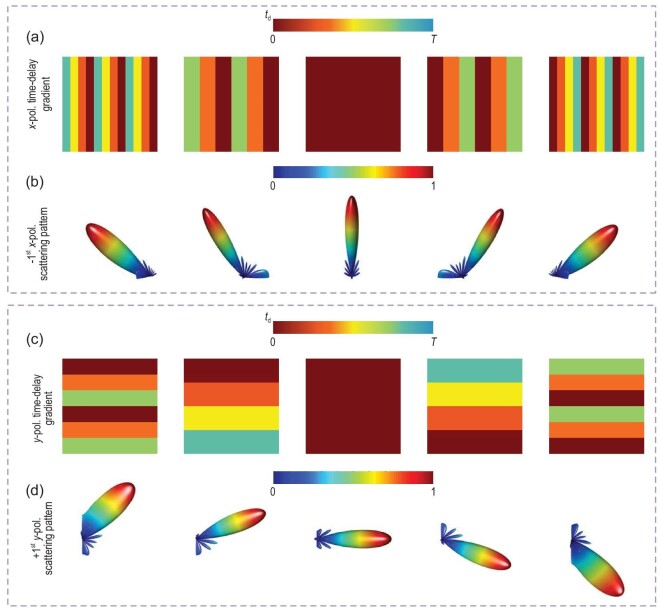
Dynamic harmonic beamforming along different polarization directions. (a) Time-delay gradients *tg*1(*x*) along the *x* polarization direction: ($\frac{{3T}}{4},\frac{T}{2},\frac{T}{4},0,\frac{{3T}}{4},\frac{T}{2},\frac{T}{4},0,\frac{{3T}}{4},\frac{T}{2},\frac{T}{4},0$), ($\frac{{2T}}{3},\frac{{2T}}{3},\frac{T}{3},\frac{T}{3},0,0,\frac{{2T}}{3},\frac{{2T}}{3},\frac{T}{3},\frac{T}{3},0,0$), (0,0,0,0,0,0,0,0,0,0,0,0), ($0,0,\frac{T}{3},\frac{T}{3},\frac{{2T}}{3},\frac{{2T}}{3},0,0,\frac{T}{3},\frac{T}{3},\frac{{2T}}{3},\frac{{2T}}{3}$) and ($0,\frac{T}{4},\frac{T}{2},\frac{{3T}}{4},0,\frac{T}{4},\frac{T}{2},\frac{{3T}}{4},0,\frac{T}{4},\frac{T}{2},\frac{{3T}}{4}$). (b) Scattering patterns of the −1st *x*-polarized harmonic with beam directions of −50.5°, −31.0°, 0°, +31.0° and +50.5° on the *xoz* plane. (c) Time-delay gradients *tg*2(*y*) along the *y* polarization direction: ($0,0,\frac{T}{3},\frac{T}{3},\frac{{2T}}{3},\frac{{2T}}{3},0,0,\frac{T}{3},\frac{T}{3},\frac{{2T}}{3},\frac{{2T}}{3}$), ($0,0,0,\frac{T}{4},\frac{T}{4},\frac{T}{4},\frac{T}{2},\frac{T}{2},\frac{T}{2},\frac{{3T}}{4},\frac{{3T}}{4},\frac{{3T}}{4}$), (0,0,0,0,0,0,0,0,0,0,0,0), ($\frac{{3T}}{4},\frac{{3T}}{4},\frac{{3T}}{4},\frac{T}{2},\frac{T}{2},\frac{T}{2},\frac{T}{4},\frac{T}{4},\frac{T}{4},0,0,0$) and ($\frac{{2T}}{3},\frac{{2T}}{3},\frac{T}{3},\frac{T}{3},0,0,\frac{{2T}}{3},\frac{{2T}}{3},\frac{T}{3},\frac{T}{3},0,0$). (d) Scattering patterns of the +1st *y*-polarized harmonic with beam directions of −47.8°, −21.7°, 0°, +21.7° and +47.8° on the *yoz* plane.

As an intuitive example of dynamic dual-harmonic beamforming along two polarization directions, we consider an anisotropic STC digital metasurface composed of 12 × 12 meta-atoms (*M* = *N* = 12) with the following configuration parameters: *f*_c_ = 2.7 GHz, T = 2 $\mu s$, and the distances between two adjacent rows and columns being 36 mm (0.324${\lambda}_{\rm{c}}$) and 25 mm (0.225${\lambda}_{\rm{c}}$), respectively, where ${\lambda}_{\rm{c}}$ is the wavelength of the incident wave. In the *x* polarization direction, we enable five groups of time-delay gradients for *tg*1(*x*), which are in turn:

$$\begin{eqnarray*}
&& \left( {\frac{{3T}}{4},\frac{T}{2},\frac{T}{4},0,\frac{{3T}}{4},\frac{T}{2},\frac{T}{4},0,\frac{{3T}}{4},\frac{T}{2},\frac{T}{4},0} \right)\!,\\
&&\left( {\frac{{2T}}{3},\frac{{2T}}{3},\frac{T}{3},\frac{T}{3},0,0,\frac{{2T}}{3},\frac{{2T}}{3},\frac{T}{3},\frac{T}{3},0,0} \right)\!,\\
&&\left( {0,0,0,0,0,0,0,0,0,0,0,0} \right)\!,\\
&&\left( {0,0,\frac{T}{3},\frac{T}{3},\frac{{2T}}{3},\frac{{2T}}{3},0,0,\frac{T}{3},\frac{T}{3},\frac{{2T}}{3},\frac{{2T}}{3}} \right)\!,\\
&&{\rm{and}}\\
&&\left( {0,\frac{T}{4},\frac{T}{2},\frac{{3T}}{4},0,\frac{T}{4},\frac{T}{2},\frac{{3T}}{4},0,\frac{T}{4},\frac{T}{2},\frac{{3T}}{4}} \right)\!;
\end{eqnarray*}$$

meanwhile, we apply the following group of time-delay gradients for *tg*2(*y*) along the *y* polarization direction, which are in turn:

$$\begin{eqnarray*}
&&\left( {0,0,\frac{T}{3},\frac{T}{3},\frac{{2T}}{3},\frac{{2T}}{3},0,0,\frac{T}{3},\frac{T}{3},\frac{{2T}}{3},\frac{{2T}}{3}} \right)\!,\\
&&\left( {0,0,0,\frac{T}{4},\frac{T}{4},\frac{T}{4},\frac{T}{2},\frac{T}{2},\frac{T}{2},\frac{{3T}}{4},\frac{{3T}}{4},\frac{{3T}}{4}} \right)\!,\\
&&\left( {0,0,0,0,0,0,0,0,0,0,0,0} \right)\!,\\
&&\left( {\frac{{3T}}{4},\frac{{3T}}{4},\frac{{3T}}{4},\frac{T}{2},\frac{T}{2},\frac{T}{2},\frac{T}{4},\frac{T}{4},\frac{T}{4},0,0,0} \right)\!,\\
&&{\rm{and}}\\
&&\left( {\frac{{2T}}{3},\frac{{2T}}{3},\frac{T}{3},\frac{T}{3},0,0,\frac{{2T}}{3},\frac{{2T}}{3},\frac{T}{3},\frac{T}{3},0,0} \right)\!,
\end{eqnarray*}$$

as illustrated from left to right in Fig. 3a (*x* polarization) and c (*y* polarization). According to Equation (8a) and (8b), we can synthesize the −1st *x*-polarized and +1st *y*-polarized beams in the directions of (${\theta }_x,{\theta }_y$) in two polarization directions, as illustrated in Fig. 3b and d, which are in the sequence of (−50.5°, −47.8°), (−31.0°, −21.7°), (0°, 0°), (+31.0°, +21.7°) and (+50.5°, +47.8°).

In the proposed modulation scheme, we use a single metasurface to achieve dynamic dual-harmonic beamforming along two polarization directions. Once the control signals of all columns and rows on the metasurface correspond to the MPSK symbols with the time-delay gradients *tg*1(*x*) and *tg*2(*y*) belonging to the modulation signal sets, as shown in Equation (S3a–d) of Supplementary Note 2, space-frequency-polarization-division multiplexed MPSK modulations with wide-angle coverages are achieved on the two polarization directions. Since the dynamic harmonic beamforming is a natural property of the STC digital metasurfaces with variable harmonic phase profiles, it should be noted that the above-mentioned space-frequency-polarization-division multiplexed modulation scheme is also applicable to other operating frequencies.

## DESIGN OF THE ANISOTROPIC STC DIGITAL METASURFACE

**Figure 4.**
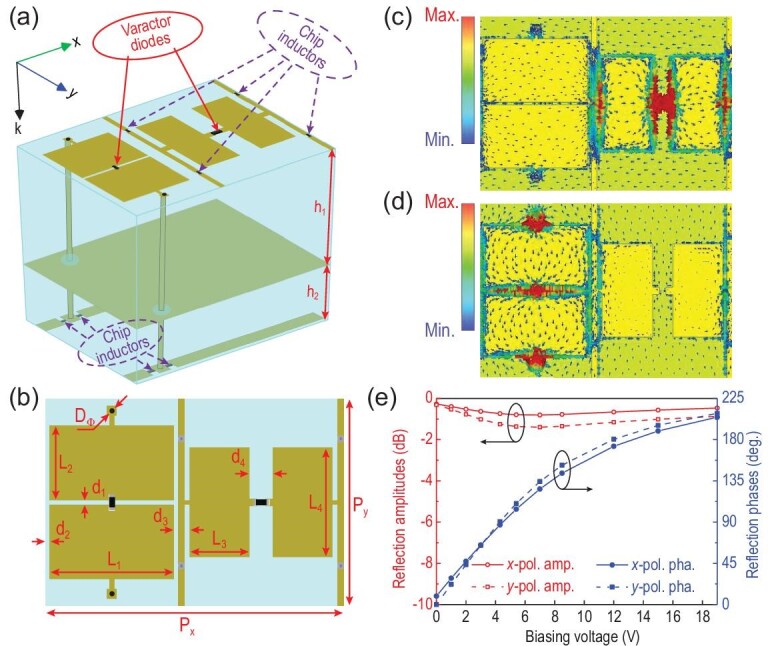
(a) Unit element of the anisotropic STC digital metasurface. (b) Top layer of the element and its geometrical parameters. Surface current distributions of the element when the metasurface is radiated by (c) *x*- and (d) *y*-polarized plane waves. (e) Relation between the *x-* and *y-*polarized reflection properties of the metasurface and the biasing voltage at 2.7 GHz.

Figure 4a shows the element diagram of the proposed anisotropic STC digital metasurface [33]. The top metal pattern on the element mainly consists of two orthogonal shared-aperture rectangular patch pairs, which are printed on a grounded F4B substrate 1 $({\epsilon }_r = 2.65,\,\,{\rm tan} \delta \ = \ 0.0015)$. Two varactor diodes (Skyworks SMV-1405) are embedded in the gap between the patch pairs, and two groups of metal strips are used to bias the varactors. To control the working states of the varactors independently, one group of metal strips is arranged on the top layer, while the other is arranged on the back of the F4B substrate bonded with substrate 1. The patch pair is connected through two metal via holes. In addition, filtering inductors (27 nH) are introduced in the biasing strips to prevent high-frequency surface currents from entering the control circuit module. Detailed geometric parameters of this programmable element are shown in Fig. 4b and Table 1.

**Table 1.** Dimensions of element in anisotropic STC digital metasurface.

Parameter	${{\boldsymbol{P}}}_{\boldsymbol{\!\! x}}$	${{\boldsymbol{P}}}_{\boldsymbol{\!\! y}}$	${{\boldsymbol{L}}}_1$	${{\boldsymbol{L}}}_2$	${{\boldsymbol{L}}}_3$	${{\boldsymbol{L}}}_4$	${{\boldsymbol{d}}}_1$	${{\boldsymbol{d}}}_2$	${{\boldsymbol{d}}}_3$	${{\boldsymbol{d}}}_4$	${{\boldsymbol{D}}}_{{\bf \Phi }}$	${{\boldsymbol{h}}}_1$	${{\boldsymbol{h}}}_2$
Value (mm)	36	25	15	9	7.2	13.2	0.4	0.5	2	2.8	0.6	5	2

Full-wave numerical simulations were conducted using the commercial software CST Microwave Studio 2016. In element simulations, infinite periodic boundary conditions were adopted, and *x-* and *y-*polarized plane waves were used to normally illuminate the element along the −*z* direction to calculate the reflection properties. In the simulation, the varactors are modeled as lumped elements with R-L-C series circuit models, and the effective parameters are listed in Supplementary Table S1 of Supplementary Note 3.

To demonstrate the EM properties of the anisotropic STC digital metasurface, Fig. 4c and d illustrate the simulated surface current distributions of the element at 2.7 GHz when radiated by the *x*- and *y*-polarized EM waves, respectively. We observe that a large number of surface currents circulate through the diode that is parallel to the incident electric field. When the working state of the diode is altered, the phase of co-polarized reflected wave component changes. However, when the working state of the other diode is changed simultaneously, owing to the minimal surface current nearby, the element cannot excite an orthogonally polarized wave. This behavior suggests the excellent polarization isolation of the element, and hence the two diodes can independently control the phases of the *x*- and *y*-polarized reflected waves. To confirm the polarization isolation, we set *x-* and *y-*polarized waves to illuminate the element simultaneously and calculate the cross-polarized reflection coefficients at different voltages. For an arbitrary combination of the biasing voltages, the cross-polarized reflection amplitude remains below −50 dB, confirming the high polarization isolation of the element.

By simulations, we can obtain large dual-polarized phase-varying range (>200°) from 2.5 to 2.7 GHz for the proposed STC digital metasurface, with an operation bandwidth of 200 MHz. Figure 4e shows the simulated reflection amplitudes and phases according to the biasing voltage of the anisotropic STC digital metasurface at 2.7 GHz; they verify the amplitude fluctuations and phase coverages of two co-polarized reflections. When the biasing voltage varies from 0 to 19 V, the phase ranges are ∼200°, and the amplitudes vary within 1 dB for both *x-* and *y*-polarized reflected waves.

Before conducting the following experiments, we fabricate the designed anisotropic STC digital metasurface using printed circuit board technology. This metasurface prototype is composed of 12 × 12 meta-atoms, and the size is 542 × 410 × 7 mm^3^. Subsequently, we measure the reflection properties of the metasurface under a frequency of 2.7 GHz. The results show that the phase coverages of *x*- and *y*-polarized reflected waves can reach 200°, and their amplitude fluctuations are within 1.5 dB. In addition, the measured result of the cross-polarized reflection amplitude is illustrated in Supplementary Fig. S2 of Supplementary Note 4. Whatever voltage value is applied to *x* and *y* polarization directions, the polarization isolation is always >20 dB. Hence, the high polarization isolation and polarization-independent phase-shifting properties of the metasurface comply with our design.

## MPSK WIRELESS COMMUNICATION SYSTEM FOR FREQUENCY-POLARIZATION-DIVISION MULTIPLEXING

Firstly, we set up an MPSK wireless communication system based on frequency-polarization-division multiplexing with the proposed anisotropic STC digital metasurface in an indoor environment. We consider 16PSK signal modulation, and we choose the phase difference of the modulation signals $\Delta \varphi = {200}^{\rm{o}}$, and the symbol period T = 2 $\mu s$. The modulation signals for the *x-* and *y*-polarization channels are given by Equation (S5a and b) in Supplementary Note 2, respectively. According to Fig. 4e, with the change of phases, the amplitudes of ${A}_x$ and ${A}_y$ will inevitably change as well. Here we choose the time-averaged ${A}_x$ and ${A}_y$ due to the small amplitude fluctuation. According to Equation (S2) in Supplementary Note 1, the *x*- and *y*-polarized harmonic energy ratios, $\frac{{{{| {{{{\boldsymbol{\vec{E}}}}}_{rx}( {{\omega }_c - {\omega }_0} )} |}}^2}}{{{{| {{{{\boldsymbol{\vec{E}}}}}_{rx}( {{\omega }_c + {\omega }_0} )} |}}^2}}$ and $\frac{{{{| {{{{\boldsymbol{\vec{E}}}}}_{ry}( {{\omega }_c + {\omega }_0} )} |}}^2}}{{{{| {{{{\boldsymbol{\vec{E}}}}}_{ry}( {{\omega }_c - {\omega }_0} )} |}}^2}}$, are both 10.9 dB, ensuring that the frequency channel of the −1st harmonic is almost the *x*-polarized wave, whereas the *y*-polarized wave is dominant in the frequency channel of the +1st harmonic.

**Figure 5.**
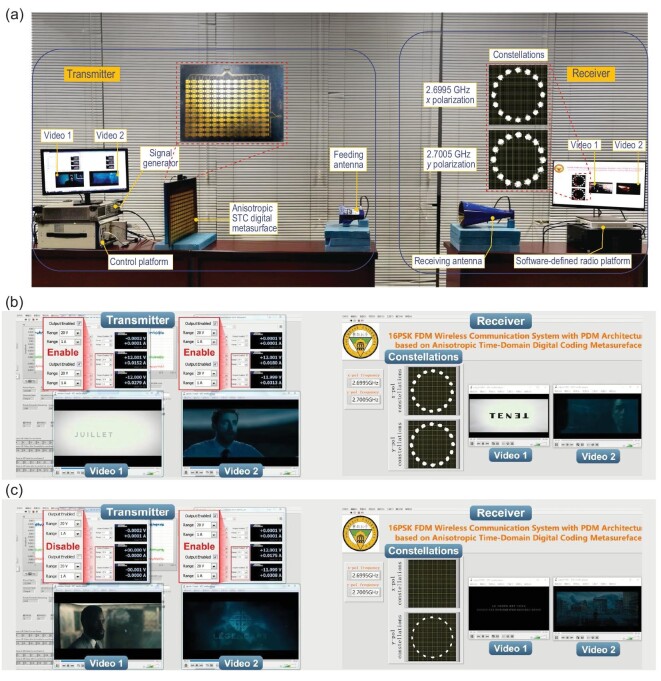
(a) Experimental set-up of the frequency-polarization-division multiplexed MPSK communication system. In the experiments, two 480p resolution (640 × 480) videos, separately modulated on 2.6995 GHz *x*-polarized and 2.7005 GHz *y*-polarized waves, are independently and synchronously transmitted from the transmitter (left) to the receiver (right). Experimental video transmission scenarios of the system when (b) dual channels are, and (c) only the 2.7005 GHz *y*-polarized channel is, enabled, in which the 16PSK constellations of the dual channels (b) and only the 2.7005 GHz *y*-polarized channel (c) are very stable. In the dual-channel situation, the transmissions of Videos 1 and 2 are very fluent; and in the later situation, only Video 2 is fluently transmitted.

Figure 5a shows the indoor wireless communication scenario, and a schematic diagram of the proposed communication system is presented in Supplementary Fig. S3 of Supplementary Note 5. During the information transmission, two independent information sources are separately encoded into two sets of binary bit streams by the transmitter. The streams are then converted into two control signal sequences to drive the anisotropic STC digital metasurface through a customized control platform. Under the radiation of a 45°-tilted horn antenna with an incident frequency of 2.7 GHz, the metasurface will simultaneously reflect the −1st *x*-polarized and +1st *y*-polarized modulated waves. At the receiver, a dual-polarized horn antenna with good polarization isolation receives the *x*- and *y*-polarized modulated signals at a distance of 3.5 m, and transmits the signals to a commercial-software-defined radio platform to recover the original information. More details on the information transmission and system equipment are available in Supplementary Note 5. In the baseband signal processing, conventional communication methods, such as least square channel estimation and zero forcing channel equalization, are used at the receiver to compensate for the channel fading.

Figure 5b shows the independent and synchronous transmissions of two 480p resolution (640 × 480) videos through the proposed frequency-polarization-division multiplexed MPSK communication system. During the video transmission, the two 16PSK constellations for the 2.6995 GHz *x*-polarized and 2.7005 GHz *y*-polarized channels remain stable, and the transmissions of Videos 1 and 2 have very good quality (see Supplementary Video 1 for details). Furthermore, as shown in Fig. 5c, when one channel (e.g. the 2.6995 GHz *x*-polarized channel) is disabled, the corresponding constellation disappears and the video recovery is interrupted, but the remaining video transmission continues without interference, showing the independent transmissions of the two videos (see Supplementary Video 1 for details). Thus, the proposed communication system operates very well, as expected with a transmission rate up to 20 Mbps.

## MPSK WIRELESS COMMUNICATION SYSTEM FOR SPACE-FREQUENCY-POLARIZATION-DIVISION MULTIPLEXING

Before experimentally demonstrating the space-frequency-polarization-division multiplexed MPSK communication system, we firstly show that the anisotropic STC digital metasurface can perform dynamic dual-harmonic beamforming along the two polarization directions. The experimental set-up is detailed in Supplementary Note 6. We assign the time-varying reflection coefficients ${\Gamma}_{\!\! xx}( t ) = {\Gamma }_x[ {t - tg1( x )} ]$ and ${\Gamma}_{\!\! yy}( t ) = {\Gamma }_y[ {t - tg2( y )} ]$ to the columns and rows of the metasurface; $tg1( x )$ and $tg2( y )$ are illustrated in Fig. 3a and c, respectively.

**Figure 6.**
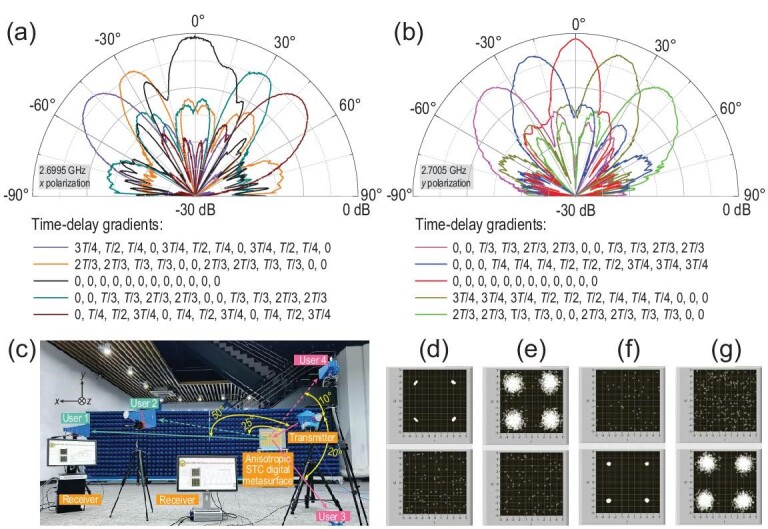
Normalized measured scattering patterns of (a) 2.6995 GHz *x*-polarized and (b) 2.7005 GHz *y*-polarized harmonics. The five groups of (color-coded) time-delay gradients are chosen as the candidates for dual-harmonic beamforming along the (a) *xoz* and (b) *yoz* polarization directions. (c) Experimental set-up of the space-frequency-polarization-division multiplexed MPSK communication system, in which Users 1 and 2 are located on the *xoz* plane, while Users 3 and 4 are located on the *yoz* plane. The four users point in different directions. (d–g) QPSK constellations for Users 1–4. We note that two perfect QPSK constellations are successfully demodulated for User 1 at the 2.6995 GHz *x*-polarized channel (d) and for User 3 at the 2.7005 GHz *y*-polarized channel (f); while two imperfect constellations are demodulated for User 2 at the 2.6995 GHz *x*-polarized channel (e) and for User 4 at the 2.7005 GHz *y*-polarized channel (g).

Figure 6a and b show the normalized far-field scattering patterns of the −1st *x*-polarized and +1st *y*-polarized waves under the five groups of time-delay gradients$\ tg1( x )$ and $tg2( y )$, and indicate that the beam directions are consistent with theoretical predictions. We observe that the harmonic beam directions of two polarizations and two frequencies can be freely manipulated, supporting the capability of the space-frequency-polarization-division multiplexing. By shifting the time-delay gradients $tg1( x )$ and $tg2( y )$, we realize dual-polarized harmonic beam scanning, in which the maximum scanning angles along the *x* and *y* polarization directions are $ \pm 50^\circ $ and $ \pm 47.8^\circ $, respectively. The scanning gains and aperture efficiencies at different scanning angles are listed in Supplementary Tables S2 and S3 of Supplementary Note 6.

We conduct a simple indoor experiment to verify the effectiveness of the proposed space-frequency-polarization-division multiplexed MPSK communication system. We use quadrature phase-shift keying (QPSK) modulation in the system for validation, where the modulation signal sets for the *x* and *y* polarization channels are given by Equation (S4a and b) in Supplementary Note 2, respectively. The other parameters are the same as those described in the simulations. The experimental set-up is shown in Fig. 6c. For the transmitter, we assign the time-delay gradients $tg1( x )$ and $tg2 (y)$ as:

\begin{eqnarray*}
&&\left( {0,\frac{T}{4},\frac{T}{2},\frac{{3T}}{4},0,\frac{T}{4},\frac{T}{2},\frac{{3T}}{4},0,\frac{T}{4},\frac{T}{2},\frac{{3T}}{4}}\right)\\
{\rm{and}}\\
&&\left({\frac{{3T}}{4},\frac{{3T}}{4},\frac{{3T}}{4},\frac{T}{2},\frac{T}{2},\frac{T}{2},\frac{T}{4},\frac{T}{4},\frac{T}{4},0,0,0}\right),
\end{eqnarray*}$$

which are embedded into the control signal sequences of the columns and rows of the metasurface, respectively. The rest of the transmitter architecture is consistent with that described in the simulations. For the receivers, four horn antennae are fixed with different angles on the *xoz* and *yoz* planes to resemble four users with different receiving directions, as shown in Fig. 6c. Here, Users 1 and 2 are represented by the *x*-polarized antennas distributed on the *xoz* plane for angles of 50° and 25° with respect to the incident path; while Users 3 and 4 are represented by the *y*-polarized antennas distributed on the *yoz* plane for angles of −20° and 10° with respect to the incident path, respectively. The four antennae are equidistant from the metasurface at 3.5 m.

Figure 6a and b demonstrate that the main beam lobe of the −1st harmonic *x*-polarized scattering pattern is approximately 50°, and that of the +1st harmonic *y*-polarized scattering pattern is approximately −20°. From Fig. 6d and f, Users 1 and 3 can demodulate nearly perfect QPSK constellation points at 2.6995 GHz for the *x*-polarized channel and at 2.7005 GHz for the *y*-polarized channel, respectively, showing excellent communication performance, since the users are located at the main beam directions of the two harmonics and polarizations. For Users 2 and 4, the corresponding constellation points become deteriorated, as illustrated in Fig. 6e and g, owing to the fact that the users are apart from the main beams and hence receive lower energies. Although the deteriorated constellation points may result in higher bit error rate (BER), the two users (2 and 4) still receive relatively good communication quality because the constellation diagrams have good shapes for QPSK. The information transmission can be seen in Supplementary Video 2, and BERs for Users 1–4 are shown in Supplementary Fig. S5 of Supplementary Note 7. This experiment indicates that the performance of space-frequency-polarization-division multiplexed MPSK communication systems is guaranteed.

## CONCLUSION AND DISCUSSION

We proposed an architecture for space-frequency-polarization-division multiplexed MPSK communications based on the anisotropic STC digital metasurface, which has been verified by two experiments. In the first experiment, wireless transmissions of two complete videos were demonstrated simultaneously in real time using the frequency-polarization-division multiplexed 16PSK scheme; while in the second experiment, the good performance of the space-frequency-polarization-division multiplexed QPSK scheme was validated with dynamic dual-harmonic beamforming along the two polarization directions. We suggest that the overall performance of the proposed system can be further enhanced by increasing the phase-varying range of meta-atoms in two polarization directions, enlarging the number of controllable meta-atoms in the metasurface, and broadening the signal bandwidth of the amplifier circuits. Compared to conventional wireless communication systems, our proposal provides low cost and high integration for general metasurface-based wireless communication systems, and more importantly it improves space utilization and channel capacities by using space-frequency-polarization-division multiplexing. Thus, the proposed theory, technology and system can be used in low-cost and simple-architecture wireless communications for multiple users.

## Supplementary Material

nwac225_Supplemental_Files
