# Supplementary material for: Space-frequency-polarization-division multiplexed wireless communication system using anisotropic space-time-coding digital metasurface
Source: Natl Sci Rev. 2022 Oct 18;9(11):nwac225. doi: 10.1093/nsr/nwac225 (PMC9701098; doi:10.1093/nsr/nwac225)
Supplement: nwac225_Supplemental_Files [file nwac225_supplemental_files.zip › Supplementary_data.docx]

Supplementary Information for

Space-frequency-polarization-division multiplexed wireless communication system using anisotropic space-time-coding digital metasurface

Jun Chen Ke^1,2,3,†^, Xiangyu Chen^4,†^, Wankai Tang^4^, Ming Zheng Chen^1,2,3^, Lei Zhang^1,2,3^, Li Wang^1,2,3^, Jun Yan Dai^1,2,3^, Jin Yang^5^, Jun Wei Zhang^1,2,3^, Lijie Wu^1,2,3^, Qiang Cheng^1,2,3,^*, Shi Jin^4,^*, and Tie Jun Cui^1,2,3,^*

**^1^** *State Key Laboratory of Millimeter Waves, Southeast University, Nanjing 210096, China*

**^2^** *Institute of Electromagnetic Space, Southeast University, Nanjing 210096, China*

**^3^** *Frontiers Science Center for Mobile Information Communication and Security, Southeast University, Nanjing 210096, China*

**^4^** *National Mobile Communications Research Laboratory, Southeast University, Nanjing 210096, China*

**^5^** *Southeast University Wuxi Campus, Wuxi 214061, China*

^†^ These authors contribute equally to this work.

^*^ Corresponding authors: [qiangcheng@seu.edu.cn](mailto:qiangcheng@seu.edu.cn); [jinshi@seu.edu.cn](mailto:jinshi@seu.edu.cn); [tjcui@seu.edu.cn](mailto:tjcui@seu.edu.cn)

**This Supplementary Information includes:**

Supplementary Notes 1 to 7

Supplementary Figures S1 to S5

Supplementary Table S1 to S3

Supplementary Equations S1 to S5

Supplementary Videos 1 to 2

Supplementary References

Supplementary Note 1: Derivation of the reflected wave $\boldsymbol{E}_{\boldsymbol{r}}\left( \boldsymbol{\omega} \right)$ for periodic $\boldsymbol{\Gamma}\mathbf{(}\boldsymbol{t}\mathbf{)}$ with linear phase

When the phase of time-varying reflection coefficient $\Gamma(t)$ is a periodically linear function, as shown in Eq. (4), the reflection spectrum distribution, $E_{r}\left( \omega\right)$, can be derived as

|  | $E_{r}\left( \omega\right)=\sum_{k=-\infty}^{+\infty} a_{k}E_{i}\left( \omega-k\omega_{0} \right),$ | (S1) |
| --- | --- | --- |

where $\omega_{0}=\frac{2\pi}{T}$ represents the modulation angular frequency, and $a_{k}$ is the *k*^th^ Fourier-series coefficient of $\Gamma\left( t \right)$ given by [1]

$a_{k}=\left| Asinc(\frac{\Delta\varphi}{2}-k\pi) \right|e^{j[\varphi_{1}+\frac{\Delta\varphi}{2}-k\pi+mod\left( \left\lfloor\frac{\Delta\varphi}{2\pi}-k \right\rfloor,2 \right)\cdot\pi+\varepsilon(2k\pi-\Delta\varphi)\cdot\pi]}$, (S2)

where $\left\lfloor\cdot\right\rfloor$ is the rounding down function, and $sinc(\cdot)$,$mod(\cdot)$, and $\varepsilon(\cdot)$ are the sinc, mod, and step functions, respectively. In accordance with the amplitude described in Eq. (S2), the +1^st^ harmonic is dominant when $\Delta\varphi>\pi$. On the contrary, if the phase of the reflection coefficient decreases linearly and satisfies $\Delta\varphi<-\pi$, the highest peak of the reflection spectrum is located at the −1^st^ harmonic.

Supplementary Note 2: Allocation strategy of the modulation signal sets for two polarization channels

To realize frequency-polarization-division multiplexed MPSK modulations, we should assign modulation signal sets $\boldsymbol{S}_{x-pol.}$ and $\boldsymbol{S}_{y-pol.}$ to the corresponding polarization channels. In general, if the MPSK modulations in both *x* and *y* polarization channels contain *m* possible symbols, the corresponding harmonics must have ${log}_{2}m$-bit phase-switchable properties. According to Fig. 2d, the phases of the +1^st^ and −1^st^ harmonics vary linearly with the time delay $t_{d}$. To this end, we set unit time delay $t_{0}=\frac{T}{m}$ to define all states of the ${log}_{2}m$-bit harmonic phases, obtaining the modulation signal sets $\boldsymbol{S}_{x-pol.}$ and $\boldsymbol{S}_{y-pol.}$ as:

$\boldsymbol{S}_{x-pol.}=\left\{ \Gamma_{xx}\left( t \right),\Gamma_{xx}\left( t-t_{0} \right),\ldots,\Gamma_{xx}[t-(m-1)t_{0}] \right\}$, (S3a)

$\boldsymbol{S}_{y-pol.}=\{\Gamma_{yy}\left( t \right),\Gamma_{yy}\left( t-t_{0} \right),\ldots,\Gamma_{yy}[t-(m-1)t_{0}]\}$, (S3b)

where

$\Gamma_{xx}\left( t-{nt}_{0} \right)=\left\{ \begin{matrix} A_{x}e^{j\Delta\varphi(1-\frac{t+T-{nt}_{0}}{T})}, 0\leq t\leq{nt}_{0} \\ A_{x}e^{j\Delta\varphi(1-\frac{t-{nt}_{0}}{T})}, {nt}_{0}<t\leq T \end{matrix} \right., n=0, 1, \ldots, m-1$, (S3c)

and

$\Gamma_{yy}\left( t-{nt}_{0} \right)=\left\{ \begin{matrix} A_{y}e^{j\Delta\varphi\frac{t+T-{nt}_{0}}{T}}, 0\leq t\leq{nt}_{0} \\ A_{y}e^{j\Delta\varphi\frac{t-{nt}_{0}}{T}}, {nt}_{0}<t\leq T \end{matrix} \right., n=0, 1, \ldots, m-1$. (S3d)

Each element in $\boldsymbol{S}_{x-pol.}$ and $\boldsymbol{S}_{y-pol.}$ corresponds to the phase state of the corresponding harmonic, which can transmit at most ${log}_{2}m$ bits of information.

For example, the corresponding $\boldsymbol{S}_{x-pol.}$ and $\boldsymbol{S}_{y-pol.}$for the frequency-polarization-division multiplexed QPSK modulations can be expressed as

$\boldsymbol{S}_{x-pol.}=\left\{ \Gamma_{xx}\left( t \right),\Gamma_{xx}\left( t-\frac{T}{4} \right),\Gamma_{xx}\left( t-\frac{T}{2} \right),\Gamma_{xx}\left( t-\frac{3T}{4} \right) \right\}$, (S4a)

$\boldsymbol{S}_{y-pol.}=\left\{ \Gamma_{yy}\left( t \right),\Gamma_{yy}\left( t-\frac{T}{4} \right),\Gamma_{yy}\left( t-\frac{T}{2} \right),\Gamma_{yy}\left( t-\frac{3T}{4} \right) \right\}$. (S4b)

For the frequency-polarization-division multiplexed 16PSK modulations, we have

$\boldsymbol{S}_{x-pol.}=\left\{ \begin{matrix} \Gamma_{xx}\left( t \right), & \Gamma_{xx}\left( t-\frac{T}{16} \right), & \Gamma_{xx}\left( t-\frac{T}{8} \right), & \Gamma_{xx}\left( t-\frac{3T}{16} \right), \\ \Gamma_{xx}\left( t-\frac{T}{4} \right), & \Gamma_{xx}\left( t-\frac{5T}{16} \right), & \Gamma_{xx}\left( t-\frac{3T}{8} \right), & \Gamma_{xx}\left( t-\frac{7T}{16} \right), \\ \Gamma_{xx}\left( t-\frac{T}{2} \right), & \Gamma_{xx}\left( t-\frac{9T}{16} \right), & \Gamma_{xx}\left( t-\frac{5T}{8} \right), & \Gamma_{xx}\left( t-\frac{11T}{16} \right), \\ \Gamma_{xx}\left( t-\frac{3T}{4} \right), & \Gamma_{xx}\left( t-\frac{13T}{16} \right), & \Gamma_{xx}\left( t-\frac{7T}{8} \right), & \Gamma_{xx}\left( t-\frac{15T}{16} \right) \end{matrix} \right\}$, (S5a)

$\boldsymbol{S}_{y-pol.}=\left\{ \begin{matrix} \Gamma_{yy}\left( t \right), & \Gamma_{yy}\left( t-\frac{T}{16} \right), & \Gamma_{yy}\left( t-\frac{T}{8} \right), & \Gamma_{yy}\left( t-\frac{3T}{16} \right), \\ \Gamma_{yy}\left( t-\frac{T}{4} \right), & \Gamma_{yy}\left( t-\frac{5T}{16} \right), & \Gamma_{yy}\left( t-\frac{3T}{8} \right), & \Gamma_{yy}\left( t-\frac{7T}{16} \right), \\ \Gamma_{yy}\left( t-\frac{T}{2} \right), & \Gamma_{yy}\left( t-\frac{9T}{16} \right), & \Gamma_{yy}\left( t-\frac{5T}{8} \right), & \Gamma_{yy}\left( t-\frac{11T}{16} \right), \\ \Gamma_{yy}\left( t-\frac{3T}{4} \right), & \Gamma_{yy}\left( t-\frac{13T}{16} \right), & \Gamma_{yy}\left( t-\frac{7T}{8} \right), & \Gamma_{yy}\left( t-\frac{15T}{16} \right) \end{matrix} \right\}$. (S5b)

Supplementary Note 3: Effective circuit model and parameters of varactor

During the EM simulations, the varactors can be equivalent to the R-L-C series circuits, as shown in Supplementary Fig. S1. The effective circuit parameter values under different reverse biasing voltages are listed in Supplementary Table S1 [2].

**Supplementary Figure S1.** The effective R-L-C series circuit of varactor.

**Supplementary Table S1.** Effective circuit parameter values under different biasing voltages.

| Biasing Voltage (V) | L (nH) | C (pF) | R (Ω) |
| --- | --- | --- | --- |
| 0 | 0.70 | 2.65 | 0.63 |
| -1 | 0.70 | 1.85 | 0.57 |
| -2 | 0.70 | 1.54 | 0.53 |
| -3 | 0.70 | 1.36 | 0.50 |
| -4 | 0.70 | 1.24 | 0.47 |
| -5 | 0.70 | 1.17 | 0.45 |
| -6 | 0.70 | 1.09 | 0.43 |
| -7 | 0.70 | 1.04 | 0.41 |
| -8 | 0.70 | 1.00 | 0.40 |
| -9 | 0.70 | 0.97 | 0.38 |
| -10 | 0.70 | 0.95 | 0.38 |
| -11 | 0.70 | 0.91 | 0.37 |
| -12 | 0.70 | 0.87 | 0.36 |
| -13 | 0.70 | 0.85 | 0.35 |
| -14 | 0.70 | 0.83 | 0.34 |
| -15 | 0.70 | 0.81 | 0.33 |
| -16 | 0.70 | 0.79 | 0.33 |
| -17 | 0.70 | 0.78 | 0.32 |
| -18 | 0.70 | 0.76 | 0.32 |
| -19 | 0.70 | 0.76 | 0.31 |

Supplementary Note 4: Measured cross-polarized reflection amplitudes for different biasing voltages under the *x* and *y* polarizations

For different biasing voltages under the *x* and *y* polarizations, we have measured the cross-polarized reflection coefficients of the metasurface, as shown in Supplementary Fig. S2. It can be seen that the cross-polarized reflection amplitude remains below -20 dB at 2.7 GHz.


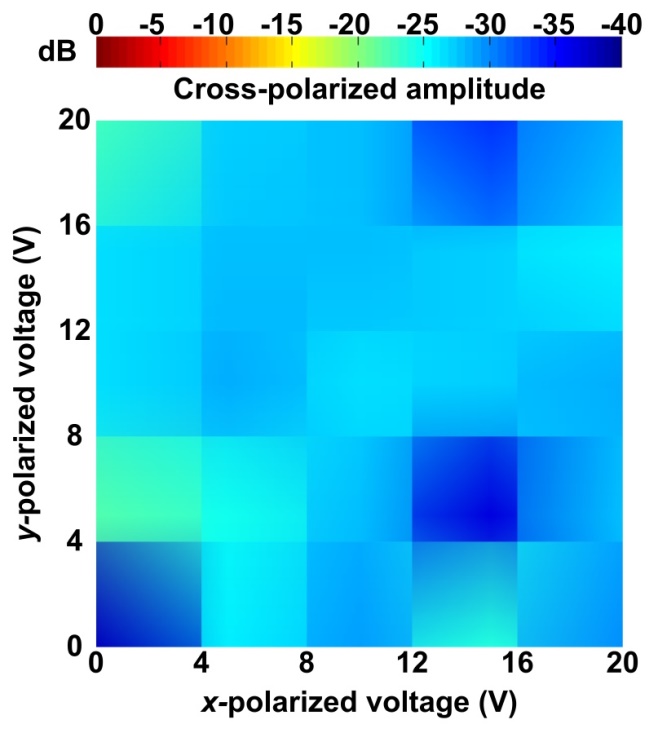


**Supplementary Figure S2.** Measured cross-polarized reflection amplitudes for different biasing voltages for *x* and *y* polarizations.

Supplementary Note 5: Frequency-polarization-division multiplexed MPSK wireless communication system

The schematic diagram of the frequency-polarization-division multiplexed MPSK wireless communication system is sllustrated in Supplementary Fig. S3. Here, Computer 1 in the transmitting end (transmitter in Fig. 5a) translates two different information sources (e.g., video files) into two sets of binary bit streams and sends them to FPGA (NI PXIe-7976R) of the control platform (NI PXIe-1082). By pre-programming the mapping relation in FPGA, the bit streams are reconstructed to symbol sequences. The two symbol sequences are separately converted into biasing voltage sequences through digital-to-analog converter (DAC, NI 5783) and external amplifier circuits. These biasing voltage sequences are then injected into the corresponding feed terminals of the anisotropic STC digital metasurface to drive the varactor diodes. Under the radiation of a 45° linearly polarized plane wave at 2.7 GHz, the metasurface reflects the −1^st^ *x*-polarized and +1^st^ *y*-polarized harmonics simultaneously. At the receiving end (receiver in Fig. 5a) placed at 3.5 *m* away, a dual-polarized horn antenna will receive the two modulated waves with different polaizations simultaneously and transmit them to the corresponding channels of a software-defined radio platform (NI USRP-2943R) with high isolation (at least 20 dB). After sequentially completing down-conversion, analog-to-digital conversion, and baseband signal processing, the modulation waves are converted to the bit streams. Finally, in Computer 2, the bit streams are reconstructed to recover the original information. During the information transmission, the interactive communication and clock synchronization among the control platform, microwave signal generator (RIGOL DSG3060), and USRP-2943R are performed using the phase stable cables.


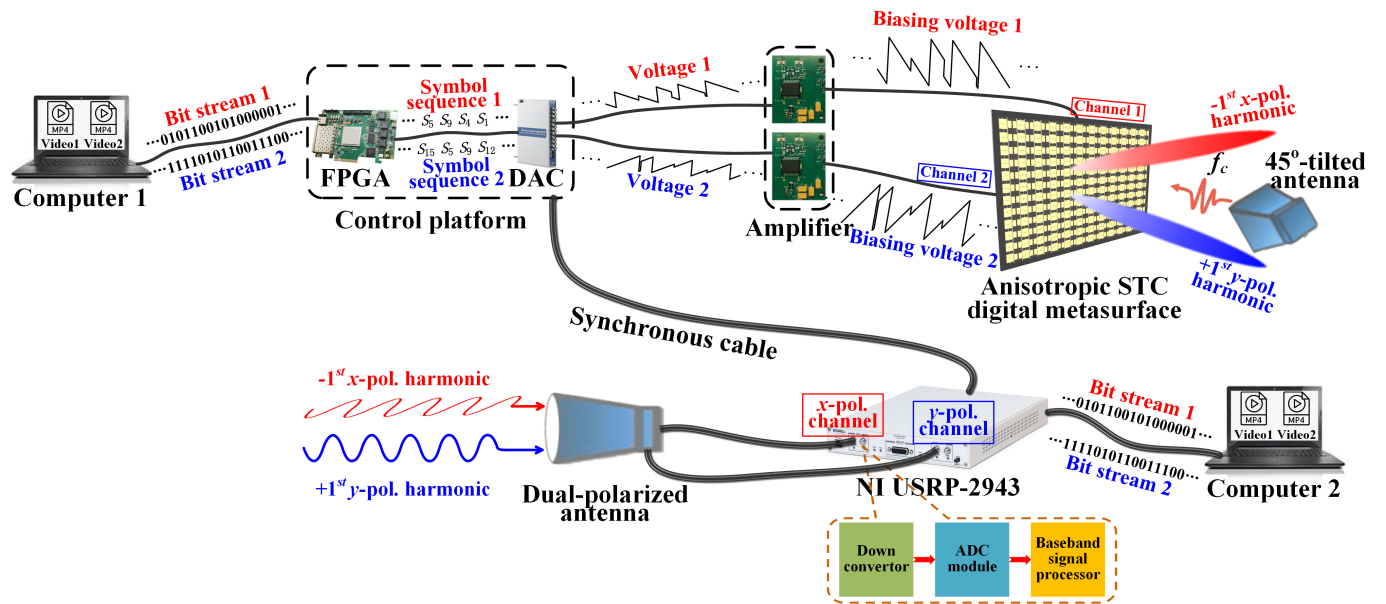


**Supplementary Figure S3.** Schematic diagram of the frequency-polarization-division multiplexed MPSK wireless communication system.

Supplementary Note 6: Dynamic dual-harmonic beamforming along two polarization directions based on the anisotropic STC digital metasurface

To experimentally validate the dynamic dual-harmonic beamforming along two polarization directions, we measure the harmonic far-field scattering patterns for different control signals. The measurement setup for beamforming on the *xoz* plane is presented in Supplementary Fig. S4a, where a microwave signal generator (Agilent E8267D) and spectrum analyzer (Agilent E4447A) are used to provide the incident wave at 2.7 GHz and receive the scattering harmonic energy, respectively, with the help of single-polarized horn antennas connected with the phase stable cables in microwave anechoic chamber. The metasurface is fixed to a swivel table rotating on the horizontal plane, and a customized control platform provides the control signal sequences for each column. All instruments are synchronized by the phase stable cables, and the metasurface sample and two horn antennas are placed at the same height. For measuring the beamforming on the *yoz* plane (Supplementary Fig. S4b), we simply flipped the metasurface by 90° and loaded the corresponding control signals towards each row of the metasurface, while the other configurations remain unchanged.


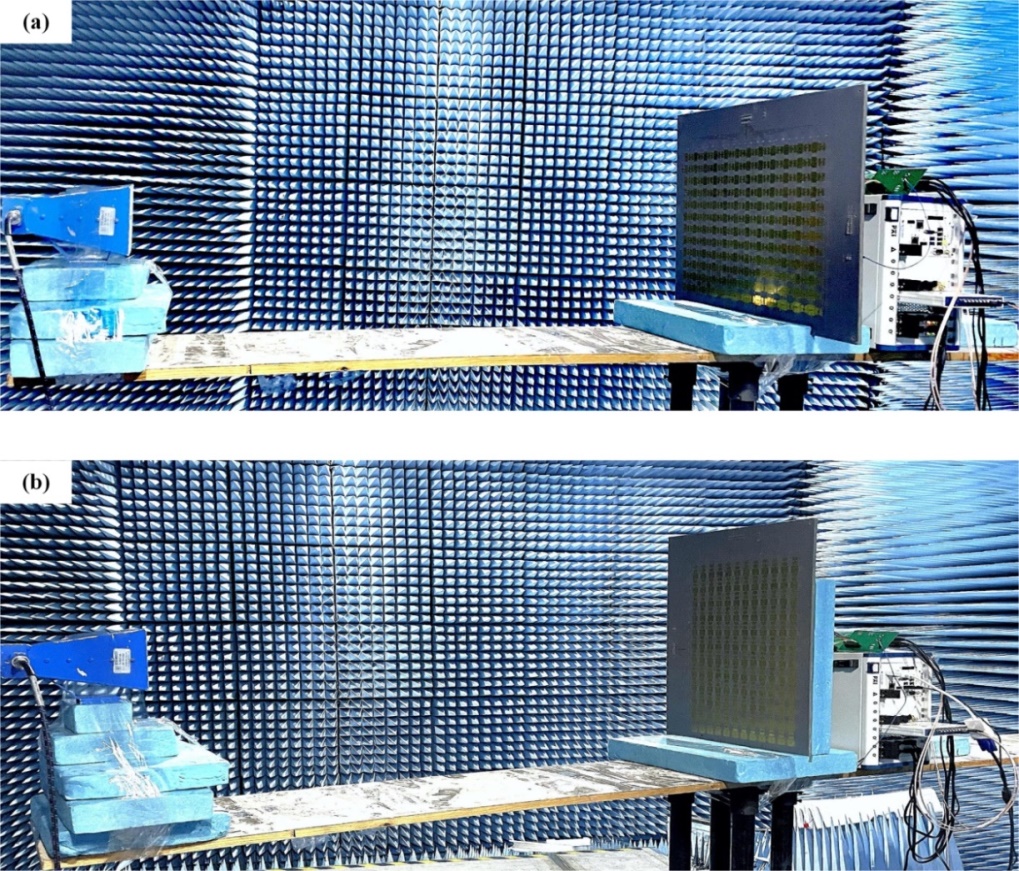


**Supplementary Figure S4.** Experimental setup for measuring the dynamic dual-harmonic beamforming along two polarization directions. Measurement setups for beamforming on the (a) *xoz*, and (b) *yoz* planes.

By shifting the time-delay gradients $tg1\left( x \right)$ and$tg2(y)$, we can realize beam scanning for dual-polarized harmonics. The comparisons of scanning gains and aperture efficiencies at different scanning angles are presented in Supplementary Tables S2 and S3, respectively. It should be noted that the feeding network occupies nearly 40% of the total area, which leads to the relatively low aperture efficiency. This can be further enhanced by optimizing the metasurface design in the future.

**Supplementary Table S2.** Scanning gains and aperture efficiencies at different scanning angles along the *x* polarization direction.

| Scanning angle (deg) | $tg1(x)$ | Scanning gain (dB) | Aperture efficiency |
| --- | --- | --- | --- |
| -50.5 | ($\frac{3T}{4},\frac{T}{2},\frac{T}{4},0,\frac{3T}{4},\frac{T}{2},\frac{T}{4},0,\frac{3T}{4},\frac{T}{2},\frac{T}{4},0$) | 17.61 | 25.5% |
| -38.1 | ($\frac{4T}{5},\frac{3T}{5},\frac{2T}{5},\frac{T}{5},0,\frac{4T}{5},\frac{3T}{5},\frac{2T}{5},\frac{T}{5},0,\frac{4T}{5},\frac{3T}{5}$) | 18.58 | 31.9% |
| -31.0 | ($\frac{2T}{3},\frac{2T}{3},\frac{T}{3},\frac{T}{3},0,0,\frac{2T}{3},\frac{2T}{3},\frac{T}{3},\frac{T}{3},0,0$) | 17.06 | 22.5% |
| -22.7 | ($\frac{3T}{4},\frac{3T}{4},\frac{T}{2},\frac{T}{2},\frac{T}{4},\frac{T}{4},0,0,\frac{3T}{4},\frac{3T}{4},\frac{T}{2},\frac{T}{2}$) | 18.20 | 29.2% |
| -14.9 | ($\frac{5T}{6},\frac{5T}{6},\frac{2T}{3},\frac{2T}{3},\frac{T}{2},\frac{T}{2},\frac{T}{3},\frac{T}{3},\frac{T}{6},\frac{T}{6},0,0$) | 19.58 | 40.1% |
| 0 | (0,0,0,0,0,0,0,0,0,0,0,0) | 20.27 | 47.1% |
| 14.9 | ($0,0,\frac{T}{6},\frac{T}{6},\frac{T}{3},\frac{T}{3},\frac{T}{2},\frac{T}{2},\frac{2T}{3},\frac{2T}{3},\frac{5T}{6},\frac{5T}{6}$) | 19.58 | 40.1% |
| 22.7 | ($0,0,\frac{T}{4},\frac{T}{4},\frac{T}{2},\frac{T}{2},\frac{3T}{4},\frac{3T}{4},0,0,\frac{T}{4},\frac{T}{4}$) | 18.20 | 29.2% |
| 31.0 | ($0,0,\frac{T}{3},\frac{T}{3},\frac{2T}{3},\frac{2T}{3},0,0,\frac{T}{3},\frac{T}{3},\frac{2T}{3},\frac{2T}{3}$) | 17.06 | 22.5% |
| 38.1 | ($0,\frac{T}{5},\frac{2T}{5},\frac{3T}{5},\frac{4T}{5},0,\frac{T}{5},\frac{2T}{5},\frac{3T}{5},\frac{4T}{5}0,\frac{T}{5}$) | 18.58 | 31.9% |
| 50.5 | ($0,\frac{T}{4},\frac{T}{2},\frac{3T}{4},0,\frac{T}{4},\frac{T}{2},\frac{3T}{4}0,\frac{T}{4},\frac{T}{2},\frac{3T}{4}$) | 17.61 | 25.5% |

**Supplementary Table S3.** Scanning gains and aperture efficiencies at different scanning angles along the *y* polarization direction.

| Scanning angle (deg) | $tg2(y)$ | Scanning gain (dB) | Aperture efficiency |
| --- | --- | --- | --- |
| -47.8 | ($0,0,\frac{T}{3},\frac{T}{3},\frac{2T}{3},\frac{2T}{3},0,0,\frac{T}{3},\frac{T}{3},\frac{2T}{3},\frac{2T}{3}$) | 16.70 | 20.7% |
| -33.7 | ($0,0,\frac{T}{4},\frac{T}{4},\frac{T}{2},\frac{T}{2},\frac{3T}{4},\frac{3T}{4},0,0,\frac{T}{4},\frac{T}{4}$) | 17.85 | 27.0% |
| -21.7 | ($0,0,0,\frac{T}{4},\frac{T}{4},\frac{T}{4},\frac{T}{2},\frac{T}{2},\frac{T}{2},\frac{3T}{4},\frac{3T}{4},\frac{3T}{4}$) | 18.15 | 28.9% |
| -12.8 | ($0,0,0,\frac{3T}{20},\frac{3T}{20},\frac{3T}{20},\frac{3T}{10},\frac{3T}{10},\frac{3T}{10},\frac{9T}{20},\frac{9T}{20},\frac{9T}{20}$) | 19.45 | 39.0% |
| 0 | (0,0,0,0,0,0,0,0,0,0,0,0) | 20.02 | 44.4% |
| 12.8 | ($\frac{9T}{20},\frac{9T}{20},\frac{9T}{20},\frac{3T}{10},\frac{3T}{10},\frac{3T}{10},\frac{3T}{20},\frac{3T}{20},\frac{3T}{20},0,0,0$) | 19.45 | 39.0% |
| 21.7 | ($\frac{3T}{4},\frac{3T}{4},\frac{3T}{4},\frac{T}{2},\frac{T}{2},\frac{T}{2},\frac{T}{4},\frac{T}{4},\frac{T}{4},0,0,0$) | 18.15 | 28.9% |
| 33.7 | ($\frac{3T}{4},\frac{3T}{4},\frac{T}{2},\frac{T}{2},\frac{T}{4},\frac{T}{4},0,0,\frac{3T}{4},\frac{3T}{4},\frac{T}{2},\frac{T}{2}$) | 17.85 | 27.0% |
| 47.8 | ($\frac{2T}{3},\frac{2T}{3},\frac{T}{3},\frac{T}{3},0,0,\frac{2T}{3},\frac{2T}{3},\frac{T}{3},\frac{T}{3},0,0$) | 16.70 | 20.7% |

Supplementary Note 7: Constellation diagrams and BERs for Users 1-4

The measured constellation and BER diagrams for Users 1-4 are presented in Supplementary Fig. S5. From the results, we find that Users 1 and 3 can get good QPSK constellations and lower BERs. However, Users 2 and 4 have deteriorated constellation and BER performance. As a consequence, better communication quality can be obtained for Users 1 and 3 than Users 2 and 4.


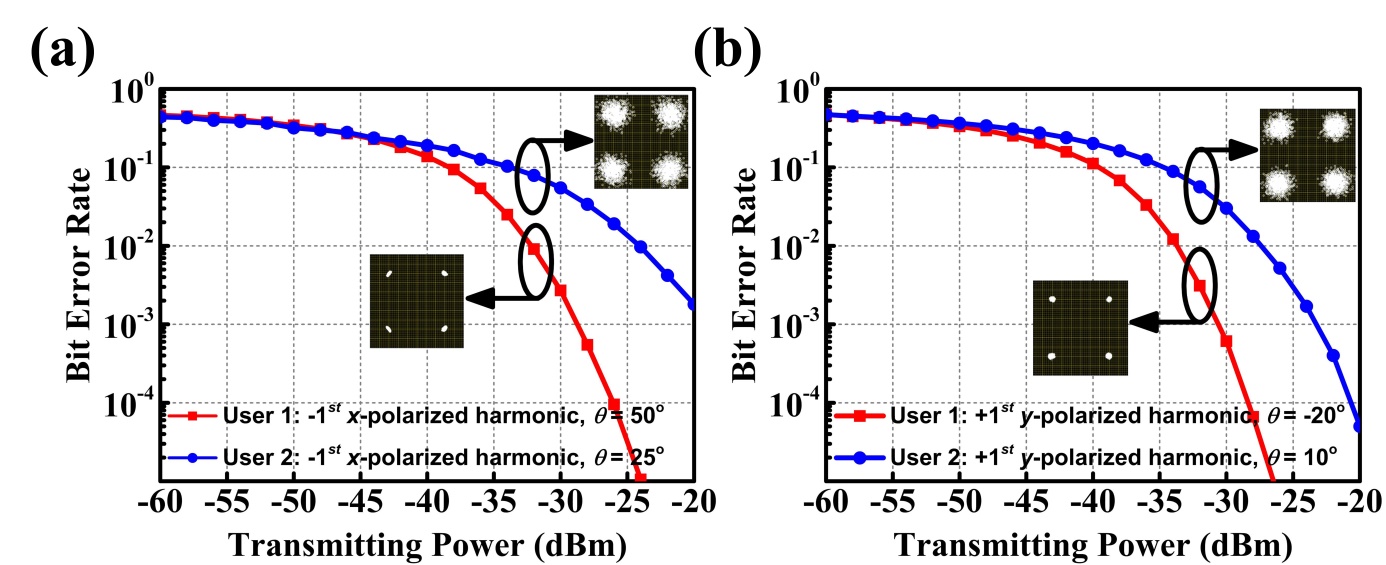


**Supplementary Figure S5.** BERs for Users 1-4 according to the measured transmitting powers by the space-frequency-polarization-division multiplexed wireless communication system, and the corresponding constellation diagrams (insets). (a) Results for Users 1 and 2. (b) Results for Users 3 and 4.

References

[1] Dai JY, Yang LX and Ke JC et al. High-efficiency synthesizer for spatial waves based on space-time-coding digital metasurface. *Laser Photon Rev* 2020; **14**: 1900133.

[2] [https://www.skyworksinc.com/-/media/SkyWorks/Documents/Products/101-200/SMV 1405_1430_Series_200068W.pdf](https://www.skyworksinc.com/-/media/SkyWorks/Documents/Products/101-200/SMV%201405_1430_Series_200068W.pdf).
